# Coculture of bacterial levans and evaluation of its anti-cancer activity against hepatocellular carcinoma cell lines

**DOI:** 10.1038/s41598-024-52699-9

**Published:** 2024-02-07

**Authors:** Walaa A. Abdel Wahab, Heba I. Shafey, Karima F. Mahrous, Mona A. Esawy, Shireen A. A. Saleh

**Affiliations:** 1 Chemistry of Natural and Microbial Products Department, Pharmaceutical and Drug Industries Research Institute, National Research Centre, Dokki, Cairo, Egypt; 2 Cell Biology Department, Biotechnology Research Institute, National Research Centre, Dokki, Cairo, Egypt

**Keywords:** Cancer, Microbiology

## Abstract

This research represents a novel study to assess how coculture affects levan yield, structure, bioactivities, and molecular weight. Among the 16 honey isolates, four bacterial strains recorded the highest levan yield. The Plackett–Burman design showed that the coculture (M) of isolates G2 and K2 had the maximum levan yield (52 g/L) and the effective factors were sucrose, incubation time, and sugarcane bagasse. The CCD showed that the most proper concentrations for maximum levan yield (81 g/L): were 130 g/L of sucrose and 6 g/f of sugarcane bagasse. Levan’s backbone was characterized, and the molecular weight was determined. G2 and K2 isolates were identified based on 16 sRNA as *Bacillus megaterium* strain YM1C10 and *Rhizobium* sp. G6-1. M levan had promising antioxidant activity (99.66%), slowed the migration activity to a great extent, and recorded 70.70% inhibition against the hepatoblastoma cell line (HepG2) at 1000 µg/mL. Gene expression analysis in liver cancer cell lines (HePG2) revealed that M levan decreased the expression of CCL20), 2GRB2, and CCR6) genes and was superior to Doxo. While increasing the expression of the IL4R and IL-10 genes. The DNA damage values were significantly increased (P < 0.01) in treated liver cancer cell lines with levan M and Doxo. The results referred to the importance of each of the hydroxyl and carboxyl groups and the molecular weight in levans bioactivities.

## Introduction

Levan is a fructan polymer and accounts for one of the most multifunctional polysaccharides. Levan yielded by the levansucrase (LSs, EC 2.4.1.10) is a glycosyltransferase that cleaves the sucrose to fructose and glucose, and then the transfructosylation process is done to form the levan with β-(2,6) glycosidic linkages. Levan has wide applications in different fields, such as food, pharmacy, and cosmetics. Previously, levan was mentioned as a prebiotic that could improve intestinal health^1^, and it has been explored in food to improve food texture. Moreover, it has many applications in the cosmetics and medical industries. Recently, levan was recorded as a strong anticancer against the pancreatic cancer cell line with no cytotoxicity on the normal retinal cell line^2^. Moreover, it played a significant role in peptic ulcer curing^3^. Also, it was recorded as an antivirus agent against pathogenic avian influenza, HPAI, H5N1, and adenovirus type 40^4^. Also, *Enterococcus faecalis* Esawy levan inhibited the New Castle Disease virus (NDV) completely^5^. Until now, the mechanism of the biological activity of levan has not been fully understood. The present study concluded that the bioactivities of G, K, and M levans depend mainly on their structures and molecular weights. It was suggested that exopolysaccharide structural characteristics, including monosaccharide residues, branching, molecular weight, glycosidic linkage, functional groups, and chemical changes, are likely to affect their antioxidant efficacy^6,7^. The molecular weight (Mw) of microbial levan is an essential index, giving this biopolymer different physiochemical and functional properties^7^. Some researchers attribute the levan anticancer activity to its immunomodulatory effect, while others attribute it to its antioxidant or prebiotic properties. At this moment, raising the production of levan has not received enough attention. Gonçalves et al.^8^ recorded that *Bacillus subtilis* NATTO levan was optimized by factorial design. Bouallegue et al.^9^ used the Plackett–Burman design for *B. subtilis* AF17. On the other hand, Ragab et al.^7^ optimized *Bacillus subtilis* M levan in a 150 L stirred tank bioreactor. In recent years, different bacterial honey isolates have been mentioned as levansucrase producers. *Bacillus subtilis* is considered the most frequent species in honey and is detected as a strong levansucrase source^4,10^. Previously, it was reported in 52 *Bacillus megaterium* from honey^11^. *Bacillus megaterium* was mentioned before as a high-activity levansucrase producer that could yield polysaccharides such as levan and dextran^12,13^. Rhizobium is a known genus of Gram-negative bacteria that plays a significant role in nitrogen fixation. Exopolysaccharides (EPS), Kdo-rich homopolymeric capsular polysaccharides, lipopolysaccharides, and cyclic (1,2)-glucans are only a few of the classes of polysaccharides that rhizobia produce. These polymers play key roles in legumes' productive symbiosis^14^.

Fermentations with mixed cultures always have at least two species in the inoculum. Mixed cultures may be made up entirely of known species or they may contain a combination of recognized and unknown species. The mixed cultures may include only one type of microbial organism, all bacteria, or they may be made up of a combination of bacteria, fungi, yeasts, or other unrelated organisms. Oriental food fermentations come across all of these combinations^15,16^.

Much research has reported that polysaccharides are a protective or preventive agent against the hepatocellular carcinoma cell line. Wu, et al.^17^ reported that gekko-sulfated polysaccharide (Gepsin) inhibited hepatocarcinoma cell growth and promoted differentiation while having a minor harmful effect on healthy liver cells. Animal and in vitro experimental studies have proposed that reactive oxygen species (ROS) are important factors in carcinogenesis. ROS-associated lesions that do not cause cell death can stimulate the development of cancer. As a force for disease prevention, there is an important balance between the free radical generation and the fighting antioxidants^18^. Antioxidants play a major role in alleviating oxidative stress by scavenging, free radicals. The migration of cancer cells is necessary for tumor development. The spread of cancer in our body is a multi-step phenomenon invaded by cancer cells surrounding tissues and blood or lymph vessels^19^.

The recent study is a unique trial designed to assess the potential of the cocultured bacteria compared to the individual cultures in levan yield and correlate the differences in structure and molecular weight for the three levans (M, G, and K) and their bioactivities. Accordingly, two methods of successive statistical design were implemented to optimize the levan yield, and the sugarcane bagasse was used instead of sugarcane to present a low-cost and eco-friendly production process. The cytotoxicity of the coculture levan (M) against HepG2: hepatocellular carcinoma was evaluated. Different studies were done to try to understand the levan mechanism. Consequently, the effect of levan M on the regulation of different genes and DNA damage was studied. Where, its mode of action was explained by liver cancer-related genes such as CCL20, GRB2, and CCR6 as well as immune-related genes IL4R and IL10, and a possible correlation between the levan in lowering the anti-migratory activity and its cytotoxicity effect in HepG2. Also, the levan M antioxidant, prebiotic and anti-migration activities were evaluated. The broad range of the hydroxyl groups and the carbonyl group C=O group of flavonoid compounds in the G levan suggested that they play an important role in levan bioactivity. On the other hand, the superiority of M in different bioactivities such as prebiotics and antioxidants could be attributed to its high molecular weights in comparison to K and G.

## Materials and methods

### Isolation of microorganisms

Isolation had been carried out using different honey sources (Group K isolated from mountain honey in Nigeria; Groups N, I, and G isolated from citrus honey, clover honey, and Marjoram honey, respectively), all of which were purchased from the Aboaouf in market, Egypt. Group V was isolated from wax honey. One hundred microliters of each honey sample were used. The plates were kept at 37 ℃ for 24 h, or until the bacterial colonies had reached a size that allowed for colony reproduction (3–5mm in diameter). The bacteria were streaked onto agar slants and kept at 4 ℃ for preservation. Microscopy was used to evaluate the purity of the isolates.

### Microorganisms’ maintenance

The isolates were regularly maintained at 80 °C in 50% (v/v) glycerol while growing on nutritional agar medium^20^ at 30 ℃.

### Levan production

The following ingredients make up the basal broth medium (BM) for the yield of levan: sucrose (80), yeast extract (1.0), K_2_HPO_4_ (1.0), and MgSO_4_ (0.2)^21^. The chosen isolates were each given a freshly made culture that was placed separately into a 250 mL Erlenmeyer flask. Each flask held 50 mL of the sample (BM).

#### Polymer yield and precipitation

In BM, the levan-producing organisms were cultivated as previously mentioned. To remove the cells, the culture was centrifuged at 5000*g*. In order to precipitate the crude levan, two liters of ice-cold, 99% ethanol were used.

### Identification of the most potent isolates

The two most potent isolates that scored the highest levan production when used as cocultures had been identified using 16s RNA primers.

#### 16S rRNA gene bacterial identification

The DNA extraction procedure for total genomic samples is based on the manufacturer’s protocol for the GeneJET Genomic DNA Purification Kit Thermo Scientific. The DNA has been purified and is ready for use in downstream applications or storage at – 20 °C^22^.

#### PCR amplification

The PCR products were amplified using 16S specific primers 27F (AGAGTTTGATCMTGGCTCAG) and 1492R (TACCTTG TTACGACTT)^23^. DreamTaq Green PCR Master Mix (2X) (K1081, Thermo Fisher, USA) was implemented for specific gene amplification according to manufacturer protocol through Creacon (Holland, Inc) Polymerase Chain Reaction (PCR) system cycler. The PCR reaction was designed with 95 ℃ for 5 min as the initial denaturing step, followed by 30 cycles of denaturing at 95 ℃ for 1 min, primer annealing at 54 ℃ for 1 min, and elongation at 72 ℃ for 90 s. Finally, extend the step at 72 ℃ for 10 min. The amplification was verified by horizontal electrophoresis on a 2% agarose gel using the Gene Ruler DNA ladder (peq GOLD 1 kb DNA-Ladder, Peqlab, VWR) according to manufacturer protocol. The gel was stained with ethidium bromide and visualized on a UV transilluminator.

### Data analysis

Gel documentation system (Geldoc-it, UVP, England) was applied for data analysis using Totallab analysis software (ww.totallab.com, Ver.1.0.1). Positive amplicons of 1500 bp were eluted from agarose gel. Resultant PCR products were purified with microspin filters and quantified spectrophotometrically. Sequence analysis was employed using the ABI PRISM® 3100 Genetic Analyzer (Micron-Corp. Korea).

### Thin-layer chromatography (TLC) analysis

HCL (0.05 N) was used to acid hydrolyze the precipitated levan. Following the addition of 2 ml of acid, each sample was heated in a boiling water bath for 2 h. Acetonitrile/water (85:15, v/v) was used in two ascents to develop thin-layer chromatography (TLC) plates. Levan on TLC plates was seen by dipping the plates into a solution of sulfuric acid (5%, v/v) in ethanol containing 1-naphthol (0.5%, w/v), followed by heating on a hot plate at 110 ℃ for 10 min^24^.

### Production optimization of levan using Plackett–Burman design (PBD)

This study has been concerned with identifying the factors affecting levan production in a parallel way using PBD^25^. Also, this study used the statistical design to apply the coculture technique by studying it as a factor and determining the most suitable isolates to act together synergistically for levan production.

Eleven components (sucrose, yeast, K_2_HPO_4_, MgSO_4_, isolates; I_1_, K_2_, V_2_, G_1_, sugarcane bagasse, banana peel, and incubation time) were selected for the study (Table 1). Each variable has two levels: high value (+ 1) and low value (− 1). A 12-run experiment was generated using the formula R = n + 1, where n is the number of variables and R is the run number.

**Table 1:** PBD for levan production.

Run	A: sucrose (g/L)	B: yeast (g/L)	C: K_2_HPO_4_ (g/L)	D: MgSO_4_ (g/L)	E:I_1_ (ml/f)	F: K_2_ (ml/f)	G: V_2_ (ml/f)	H: G_1_ (ml/f)	J: sugarcane (g/f)	K: banana peel (g/f)	L: incubation time (hours)	Levan weight (g/L)	Levan predicted value
1	0	1	1	0.1	4	4	4	1	2.5	1	48	8.5	8.83
2	120	0.5	1	0.2	4	0	0	1	5	1	48	37	36.67
3	0	0.5	0.25	0.1	0	0	0	1	2.5	1	24	40	40.27
4	120	1	0.25	0.2	4	4	0	1	2.5	2	24	28.3	28.63
5	120	1	1	0.1	0	0	4	1	5	2	24	21	20.67
6	120	1	0.25	0.1	0	4	0	4	5	1	48	52	51.73
7	120	0.5	0.25	0.1	4	0	4	4	2.5	2	48	27.3	27.57
8	0	0.5	1	0.1	4	4	0	4	5	2	24	10	9.73
9	120	0.5	1	0.2	0	4	4	4	2.5	1	24	21.4	21.73
10	0	1	1	0.2	0	0	0	4	2.5	2	48	4	4.27
11	0	1	0.25	0.2	4	0	4	4	5	1	24	2.2	1.87
12	0	0.5	0.25	0.2	0	4	4	1	5	2	48	10.9	10.63

N.B. I_1_, K_2_, V_2_, and G_4_ are bacterial honey isolates.

The PB experimental design is built on the first-order model shown below.

1 $${\text{Y}}=\upbeta 0+{\sum }_{i=0}^{k}Bi Xi,$$

where X_i_ is the level of the independent variable, Y is the response (levan production), B_0_ is the model intercept, B_i_ is the linear coefficient, and k is the total number of variables involved.

The response variable for each experiment was the average production, and trials were conducted in triplicate. Standard mistakes in a balanced design should be comparable to one another. Reduced standard errors are preferable. VIF and Ri2 should both be set to 1.0. A high Ri2 suggests that sentences are interconnected.

Further optimization trials evaluated the factors that, according to regression analysis, showed a significant impact on production (95% confidence level, Prob > F 0.05).

### CCD for factors affecting levan production

Following PBD’s estimation of the most important elements for levan production, CCD is used to identify the best concentration of these three factors (sucrose, incubation time, and sugarcane bagasse) that scored the highest positive effect.

The five coded levels (− 2, − 1, 0, + 1, + 2) for the study’s variables led to a total of 20 experimental trials, including 8 factorial design trials, 6 axial point trials, and 6 replications of the central point trials (Table 2). The optimal setting established in the prior studies was maintained for all other factors. Using a multiple regression technique, the following second-order polynomial was used to represent the results of CCD:

$${\text{Y}}\, = \,\beta 0\, + \,\Sigma \, \beta {\text{i Xi}}\, + \,\Sigma \, \beta {\text{ii X2i}}\, + \,\Sigma \, \beta {\text{ij Xi Xj}}.$$

**Table 2:** CCD for levan production.

Run	Sucrose conc. (g/L)	Incubation time (hours)	Sugar cane (g/f)	Levan weight (g/L)	Levan predicted value
1	95	36	4.25	26.00	27.28
2	130	24	6.00	81.50	66.41
3	130	24	2.50	26.00	25.96
4	95	36	4.25	23.70	27.28
5	36.15	36	4.25	10.30	6.35
6	95	36	4.25	26.30	27.28
7	60	48	6.00	15.20	9.21
8	95	56.18	4.25	27.20	24.49
9	153.86	36	4.25	40.30	48.20
10	95	36	1.31	23.00	17.65
11	60	24	6.00	16.50	15.88
12	95	36	7.19	29.00	36.90
13	130	48	2.50	31.40	26.00
14	130	48	6.00	42.00	40.50
15	60	48	2.50	17.70	26.76
16	95	36	4.25	25.10	27.28
17	95	36	4.25	22.30	27.28
18	95	15.82	4.25	23.00	30.06
19	95	36	4.25	27.00	27.28
20	60	24	2.50	12.00	7.48

Y and β0 are the predicted response and the intercept term, respectively. βi means the linear coefficients, and βii is the quadratic coefficients. βij means the interactive coefficients, and xi and xj represent independent variables coded.

### Validation of the model

Two experimental combinations were run under the conditions provided by the model to test the model’s validity. The outcomes were compared to the predictions.

### Statistical analysis

The analysis of variance approach was used to statistically analyze the model (ANOVA).

### Characterization of *Bacillus megaterium* OP315322 and *Rhizobium* sp.OP363928 and the coculture levans (K, G, M respectively).

#### Analysis using the Fourier-transform infrared (FTIR) spectrometer

The infrared spectra were evaluated using Fourier transform infrared spectroscopy (FTIR-8300, Shimadzu, Japan). The FTIR spectra’s wavelength range was 4000 to 400 cm.

#### ^1^H and ^13^C NMR analysis

Analyzing 1H and 13C NMR spectra makes it possible to count the distinct C atoms in a molecule and use the chemical shifts table to find the functional groups that are present in a levan molecule. Levan sample was dissolved in distilled water, and measurements were made with a Nawah Scientific Company JEOL-GX-500 NMR spectrophotometer set at 40℃. Levan’s spectrum was determined at 100 MHz for the 13C NMR and 400 MHz for the 1H NMR. 2.5 s for 1H NMR and 3.5 s for 13C NMR were the delay times.

#### Determination of molecular weight

Levans were made at various concentrations, and using a *U*-shaped Ostwald viscometer, flow times were recorded at 30 °C for equal quantities of each concentration. The same volume of distilled water's flow rate served as the control. As a result, the specific viscosity/C (sp) was calculated. As a result, a straight line can be drawn when plotting the concentration of levans and oligosaccharides (C) against the intrinsic viscosity (C); the intercept with the Y ordinate provides the value of (η)^26^. The following equation was used to calculate the molecular weight:

$${\text{Mol}}.{\text{ wt}}. \, = { 1}.{42 } \times { 1}0{6}\left( {{\upeta }^{{2}} } \right).$$

### Prebiotic activity (in vitro)

With few modifications, this was carried out in accordance with Tadayoni et al.^27^.

. The prebiotic cultures (*Lactobacillus plantarum* KU985432 and *Bifidobacterium lactis*) were inoculated in MRS broth supplemented with the various types of levan, (G, K, M) (1%) or water as a control and incubated for 24 h at 37 ℃ in anaer/obic circumstances. Bacterial growth at 600 nm was measured spectrophotometrically to determine the percentage of growth in the control, which was taken as 100%.

### Antioxidant activity

The three different levans kinds or water (as a control) were added in equal proportions (500 l) and forcefully mixed before being incubated at 37 ℃ in the dark for 1 hour^28^. At 517 nm, the mixture’s absorbance was determined spectrophotometrically. The scavenging activity was determined as mentioned below:

$${\text{Scavenging activity }}\% \, = \,{1}\, - \,\left( {{\text{As}}\, - \,{\text{Ab}}} \right)/{\text{ Ac}}\, \times \,{1}00.$$

where Ab, Ac, and As represent, the absorbance of the sample and the blank (ethanol and sample), the control (DPPH and deionized water), and the (DPPH and sample), respectively. Ascorbic acid, which has 100% antioxidant activity, was utilized as a reference drug for antioxidants.

### Anti-migrating activity (wound healing assay)

HepG2: Nawah Scientific Inc. provided the cell line for hepatocellular carcinoma (Mokatam, Cairo, Egypt). Cells were maintained at 37 ℃ in a humidified, 5% (v/v) CO_2_ atmosphere in Dulbecco Modified Eagles Medium (DMEM) media supplemented with 100 mg/mL of streptomycin, 100 units/mL of penicillin, and 10% heat-inactivated fetal bovine serum.

For the scratch wound experiment, cells were seeded at a density of 2 × 105 per well onto a coated 12-well plate and cultivated overnight in 5% FBS-DMEM at 37 ℃ and 5% CO_2_. The confluent monolayer was scratched horizontally the following day. The plate was carefully cleaned with PBS, the control wells were refilled with fresh medium, and the drug wells were treated with fresh media containing the levan sample. At the designated times, pictures were captured with an inverted microscope. Between each time point, the plate was incubated at 37 ℃ and 5% CO_2_. The MII Image View program, version 3.7, was used to examine the captured images, which are shown below.

#### Wound width

The average distance between the edges of the scratches was used to calculate the wound width, which decreases as cell migration is stimulated. The outcomes are presented as means with standard deviations.

### Anticancer activity (cytotoxicity assay) for levan type M

#### Cell culture

Nawah Scientific Inc. provided the HepG2: hepatocellular carcinoma samples (Mokatam, Cairo, Egypt). Cells were kept alive in DMEM media supplemented with 10% heat-inactivated foetal bovine serum, 100 mg/mL streptomycin, and 100 units/mL penicillin in a humidified, 5% (v/v) CO_2_ atmosphere at 37 ℃.

#### Cytotoxicity assay

The SRB assay was used to measure cell viability. 96-well plates were used to incubate aliquots of a 100 L cell suspension (5 × 10^3^ cells) for 24 h in full medium. Another aliquot of 100 L of medium containing levan at varying concentrations was applied to the cells. Cells were fixed by changing the medium with 150 l of 10% TCA and incubating at 4 ℃ for an hour after 72 h of levan exposure. When the TCA solution was withdrawn, distilled water was used to wash the cells five times. Seventy liters of 0.4% w/v SRB solution were added in aliquots, and they were then incubated at room temperature for 10 min in a dark area. Plates were cleaned with 1% acetic acid three times before being let air dry overnight.

Then, 150 μL of TRIS (10 mM) was added to dissolve the protein-bound SRB stain; the absorbance was measured at 540 nm using a BMG LABTECH®-FLUOstar Omega micro-plate reader (Ortenberg, Germany)^29^.

### Quantitative real time PCR method

#### RNA isolation and reverse transcription (RT) reaction

“Quantitative real-time PCR (qRT-PCR) assay was performed to quantify the change in expression of several key immune and genotoxicity-related genes within the treated cells. Total RNA was isolated from liver cancer cell lines (HePG2) samples using the RNeasy. Mini Kit (Qiagen, Hilden, Germany) in addition to the DNaseI (Qiagen) digestion step. Isolated total RNA was re-suspended in DEPC-treated water and measured photospectrophotometrically at 260 nm after being treated with one unit of RQ1 RNAse-free DNAse (Invitrogen, Germany) to breakdown DNA residues. The ratio of 260 to 280 nm, which ranged between 1.8 and 2.1, was used to determine the purity of total RNA. Ethidium bromide-stain examination of the 28S and 18S bands by formaldehyde-containing agarose gel electrophoresis further ensured integrity. Reverse transcription (RT) aliquots were used right away; otherwise, they were kept at − 80℃.

Utilizing the RevertAidTM First Strand cDNA Synthesis Kit, whole Poly(A) + RNA extracted from liver cancer cell lines (HePG2) was reverse transcribed into cDNA in a volume of 20 (μl) (Fermentas, Germany). A master mix was utilized along with 5 g of total RNA. 50 mM MgCl_2_, 10 × RT buffer (50 mM KCl, 10 mM Tris–HCl, pH 8.3), 10 mM of each dNTP, 50 M oligo-dT primer, 20 IU ribonuclease inhibitor (50 kDa recombinant enzyme to block RNase activity), and 50 IU MuLV reverse transcriptase made up the master mix.

Each sample mixture was centrifuged for 30 s at 1000*g* before being transferred to the thermocycler. The RT reaction was carried out at 25 ℃ for 10 min, then at 42 ℃ for 1 h, and finally at 99 ℃ for 5 min. The reaction tubes containing the RT preparations were then flash-cooled in an ice chamber before being used for quantitative Real Time-polymerase chain reaction amplification of cDNA (qRT-PCR).

### Real time- PCR (qPCR)

#### Gene expression analysis

Applied Biosystems' step OneTM real-time PCR system^30^ was used to determine the cDNA copy number of liver cancer cell lines (HePG2) (Thermo Fisher Scientific, Waltham, MA USA). The following ingredients were used to set up the PCR reactions: 12.5 L of 1 SYBR® Premix Ex TaqTM (TaKaRa, Biotech. Co. Ltd.), 0.5 L of 0.2 M sense primer, 0.5 L of 0.2 M antisense primer, 6.5 L of distilled water, and 5 L of cDNA template. The reaction programmed has three steps. Three minutes were spent in the first phase at 95.0 ℃. The second step included 40 cycles separated into three steps: step (a) at 95 ℃ for 15 s; step (b) at 55 ℃ for 30 s; and step (c) at 72.0 ℃ for 30 s. The third stage was broken down into 71 cycles that began at 60.0 ℃ and rose by around 0.5 ℃ every 10 s to a maximum of 95.0 ℃. To evaluate the quality of the employed primers, a melting curve analysis was carried out at 95.0 ℃ at the conclusion of each qRT-PCR. Each experiment included a distilled water control. The sequences of specific primers for the liver cancer-related genes such as CCL20^31^ (chemokine (C–C motif) ligand 20, GRB2^32^ (growth factor receptor-bound protein 2), and CCR6^33^ (chemokine (C–C motif) receptor 6), as well as immune-related genes such as IL4R^34^ (interleukin 4 receptor) and IL10^35^ (interleukin 10), were designed and listed (data not shown)**.** The relative quantification of the target to the reference was determined by using the 2 − ΔΔCT method^31,36,31^.

#### DNA damage in breast cancer cell lines (MCF-7) using the comet assay

According to Olive et al.^38^ liver cancer cell lines (HePG2) were used to detect DNA damage using the comet test (1990)^39^. After being trypsinized to create a single cell suspension, 1.5 104 cells were quickly pipetted onto a microscope slide that had already been coated. The cells were then embedded in 0.75% low-gelling-temperature agarose.

In 0.5% SDS, 30 mM EDTA, and pH 8.0, samples were lysed for 4 h at 50 °C. Samples were electrophoresed for 25 min at 0.6 V/cm before being stained with propidium iodide after being rinsed in Tris/borate/EDTA buffer, pH 8.0, overnight at room temperature. In order to determine the tail moment, DNA content, and percentage of DNA in the tail, 150 distinct comet photographs from each sample were examined under a fluorescence microscope using a CCD camera. The percentage of comet-like cells with DNA damage was calculated for each sample using an examination of roughly 100 cells.

Depend on perceived comet tail length migration and the relative amount of DNA in the nucleus, randomly selected nonoverlapping cells were visually graded on a scale of 0–3 (class 0 = no detectable DNA damage and no tail; class 1 = tail less than the nucleus diameter; class 2 = tail between 1 and 2 the nuclear diameter; and class 3 = tail longer than 2 the diameter of the nucleus)^39^.

## Results and discussion

### Isolation and identification of the most potent isolates

**Figure 1:**
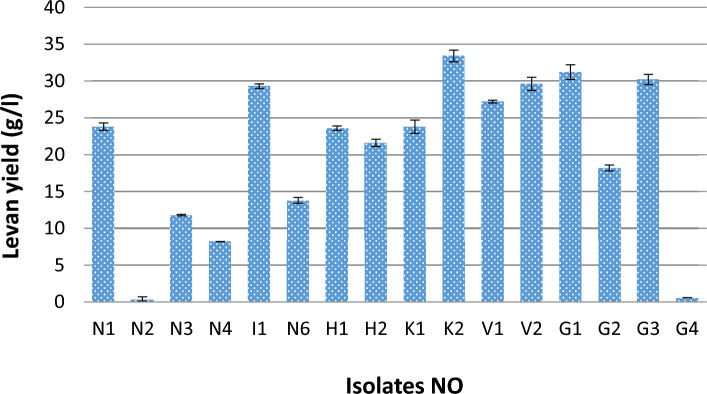
Screening for levan production by different honey isolates.

The isolation from different honey sources accounted for 16 bacterial strains, all of which were levan producers (Fig. 1). Numerous microorganisms yield levan as their primary metabolic by-product in anoxic conditions, including *B. subtilis*, *B. lentus, Z. mobilis*, *Lactobacillus sanfranciscensis, P. brassicacearum,* and *Microbacterium laevan iformans*^40^. Consequently, honey’s osmophilic and anoxic nature has recommended it in the past few years as a new reservoir for bacterial levansucrases^4,41,42^. Levan has also been shown to aid soil-dwelling bacteria in adapting to severe osmotic stress and in the production of biofilms. It has also been shown to help bacteria endure salt and desiccation as well as form cell aggregates on inorganic surfaces^43^. The four most potent isolates (I_1_, K_2_, V_2_, and G_1_) had been implemented in the optimization design (PBD) as mentioned below. The two isolates (K_2_ and G_1_) that produced the highest quantity of levan had been identified genetically using 16s RNA sequencing and submitted to the Genbank; their accession numbers are (*Bacillus megaterium* OP315322 and Rhizobium sp. OP363928). A rare study was mentioned about *Bacillus megaterium* levan^11^. Exopolysaccharides (EPS) are formed by different rhizobial species, but to our knowledge, no one has reported it as a levan producer. On the contrary, it was reported that glucose and galactose make up the majority of the biopolymers made from rhizobial species, with small amounts of other neutral and acidic monosaccharides being present^44^.

### Optimization of levan production by multi-factorial experiments

In this study, a sequential optimization technique (PBD, followed by CCD) was used. The preliminary design was used to test the variables influencing levan production. In this design, we also wanted to see if the coculture technique improves or degrades levan production by using the different isolates as independent factors to see if applying them together will be an optimizing factor in levan production or not. The second phase was optimizing the concentration of the elements that had a positive effect on levan formation.

#### Estimation of the factors affecting levan productivity using (PBD)

**Figure 2:**
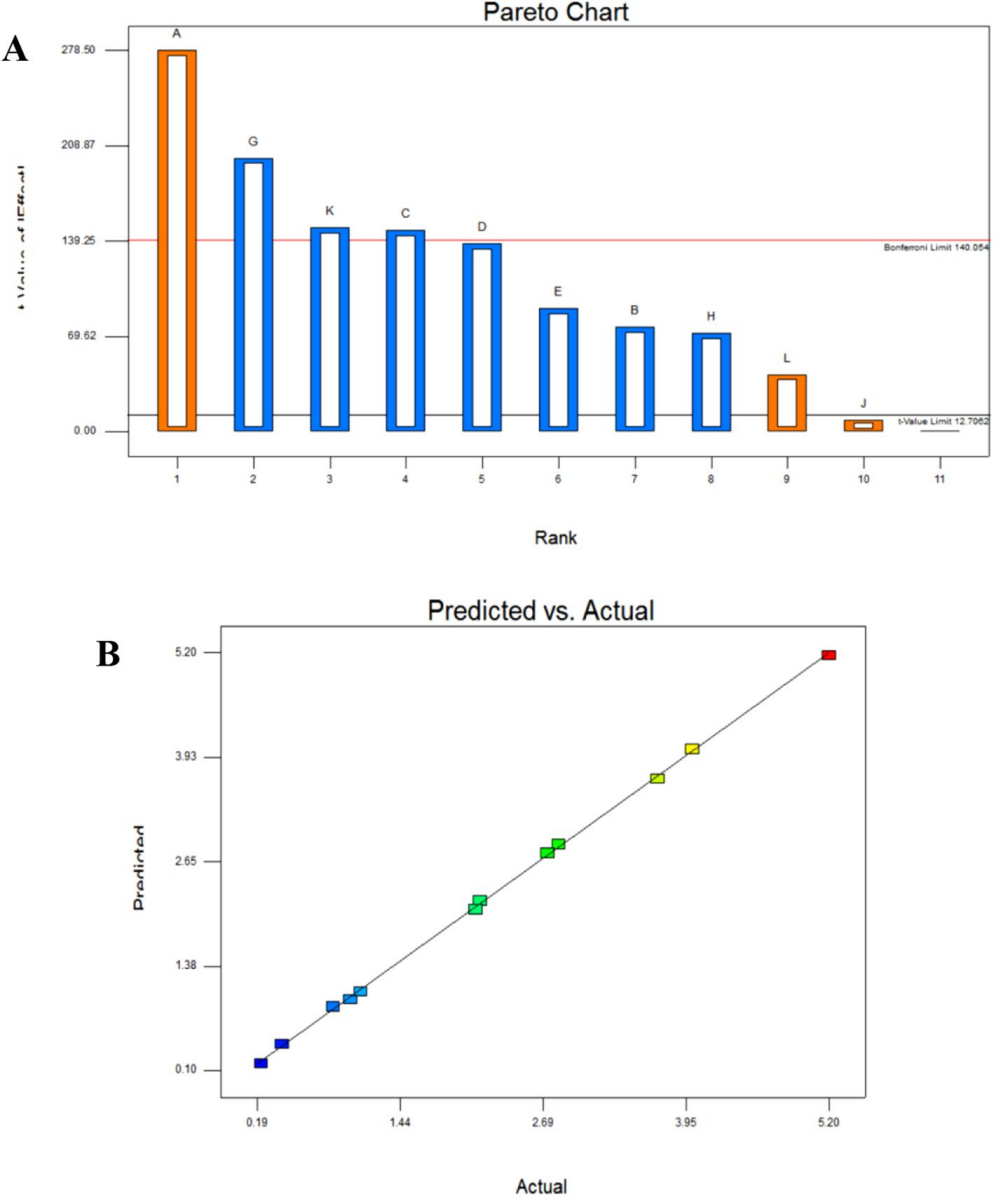
(**A**) Pareto chart showing positive and negative factors affecting levan production optimization using PBD. (**B**) Predicted vs actual graph of levan PBD showing the closure between their values emphasizing the model significance.

To describe the relationships between various medium components, the (PB) design was first created. For the optimization process, eleven parameters (A–L), including culture conditions and medium components, were chosen. Levan output was calculated as g/L and is shown in Table 1. Levan’s response showed a significant variance of (2.2–52) grams. Table 3 displays the results of the PBD's ANOVA analysis. The model's F-value of 545.20 suggests that it is significant. There is only a 0.18% chance that an F-value this large could occur due to noise. The model terms are considered significant when "Prob > F" values are less than 0.05. In this instance, all of the variables are effective and essential (i.e., they affect levan production). This circumstance, which is uncommon in statistical design research, makes evident the ideal and precise selection for the elements under investigation. It is worth noting that a significant factor is not necessarily a positive one, so although all the tested factors were significant, only three of them had a positive effect on levan production, as shown in the Pareto chart (Fig. 2A). The highest trial in levan production resulted from coculture between K2 and G1 isolates, *Bacillus megaterium *OP315322 and *Rhizobium* sp. OP363928, and was higher than each isolate's production separately, indicating that they acted synergistically to produce levan (coculture procedure).

**Table 3:** ANOVA for PBD for levan production.

Source	Sum of squares	df	Mean square	F Value	p-value, prob > F	
Model	26.82383	9	2.980426	545.1999	0.0018	Significant
A-sucrose	10.34163	1	10.34163	1891.762	0.0005	
B-yeast	0.7803	1	0.7803	142.7378	0.0069	
C-K_2_HPO_4_	2.8812	1	2.8812	527.0488	0.0019	
D-MgSO_4_	2.520833	1	2.520833	461.128	0.0022	
E-I_1_	1.08	1	1.08	197.561	0.0050	
G-V_4_	5.333333	1	5.333333	975.6098	0.0010	
H-G_1_	0.6912	1	0.6912	126.439	0.0078	
K-banana peel	2.960133	1	2.960133	541.4878	0.0018	
l-incubation time	0.2352	1	0.2352	43.02439	0.0225	

R^2^ = 0.999, adjusted R^2^ = 0.997, predicted R^2^ = 0.985, C.V. % = 3.378, Adeq precision = 73.88.

The R^2^ coefficient was 0.999, indicating that the model explains the observed variability very well. The "predicted R^2^" of 0.9853 and the "adjusted R^2^" of 0.9978 are reasonably in agreement; the difference is less than 0.2. The low coefficient of variation readings (CV = 3.378%) also supported the validity of the trials conducted. The CV is the standard error of the estimate compared to the mean value of the observed response, and a model is thought to be reasonably applicable if the CV is less than 10%. Additionally, the "Adeq Precision" ratio of 73.882 indicates a sufficient signal, refers to the model's significance, and can be used to move around in the design space.

Sucrose, incubation time, and sugarcane bagasse were the only positively influencing factors in the medium, according to the Pareto chart (Fig. 2A) for experimental data analysis. In fact, the positive effect of sucrose on levan construction is widely anticipated. Because levan is formed by assembling fructose units produced by the hydrolysis of sucrose into glucose and fructose. Many authors (Gonzalez‑Garcinuno et al.^45^, Hou et al.^46^ considered sucrose concentration as one of the factors controlling not only the production of levan but also its molecular weight. Levan production using sugarcane bagasse is very precious, as it helps recycle this waste into a valuable product. Researchers’ trials to use sugarcane bagasse started a long time ago. Han & Watson^47^ produced levan from *Bacillus polymyxa* (NRRL-18475) using sugarcane bagasse and beet molasses. Borsari et al.^48^ studied the effect of sugarcane bagasse juice on levan production using a 23-factorial design. Contrarily, Borsari et al.^48^ reported that sugarcane bagasse was not significant for levan production by *Zymomonas mobilis*. Kim & Day^49^ determined the chemical composition of sugarcane bagasse as 42% cellulose, 25% hemicellulose, and 20% lignin. However, the sugarcane bagasse culture generates 140 kg of bagasse as residue for every ton of sugarcane. As a result, this manufacturing technique allows for waste disposal in an environmentally friendly manner, which is critical for allowing our planet to breathe again.

The following is the final equation in terms of real factors:

$${\text{Levan weight}}\, = \,{6}.{65333}\, + \,0.0{15472 }*{\text{ Sucrose}} - {1}.0{2 }*{\text{ Yeast}} - {1}.{3}0{667 }*{\text{ K2HPO4}} - {9}.{16667 }*{\text{ MgSO4}} - 0.{15 }*{\text{I1 }} - 0.{33333}*{\text{ V4}} - 0.{16 }*{\text{ G1}} - 0.{99333 }*{\text{ Banana peel}}\, + \,0.0{11667 }*{\text{ Incubation Time}}.$$

The levan production’s closure between actual and predicted values (Fig. 2B) underscores the design’s great relevance.

#### CCD regression model analysis for levan production optimization

To establish the ideal concentration for the three most important variables (sucrose, sugarcane bagasse, and incubation period), the experiment underwent the second stage, the CCD (second-order model). Table 2 displays the design matrix and associated experimental data for the three independent variables. Table 4 displays the design’s examination. Levan production was significantly impacted by the linear coefficients (A & C) at P < 0.05. Similarly, the interaction terms (AC & BC) were significant. On the other hand, none of the quadratic coefficients were significant. The following is the second-order polynomial equation that the multiple regression analysis produced:

$${\text{Levan weight}}\, = \, - {4}.{9}0{716}\, + \,0.0{212}0{9}* \, *{\text{ Sucrose concentration}}\, + \,0.{226336 }*{\text{ Incubation time}}\, + \,0.{196553 }*{\text{ Sugarcane bagasse }}{-} \, 0.00{115 }*{\text{ Sucrose concentration }}*{\text{ Incubation time}}\, + \,0.0{13}0{82 }*{\text{ Sucrose concentration }}*{\text{ Sugarcane bagasse }}{-} \, 0.0{3}0{89 }*{\text{ Incubation time }}*{\text{ Sugarcane bagasse}}.$$

**Table 4:** ANOVA for CCD for levan production.

Source	Sum of squares	df	Mean square	F Value	p-value, prob > F	
Model	36.35779	6	6.059632	11.77158	0.0001	Significant
A-sucrose concentration	21.15003	1	21.15003	41.08655	< 0.0001	
B-incubation time	0.375203	1	0.375203	0.728879	0.4087	
C-sugar cane	4.476722	1	4.476722	8.696587	0.0113	
AB	1.852813	1	1.852813	3.599317	0.0802	
AC	5.136013	1	5.136013	9.977339	0.0075	
BC	3.367013	1	3.367013	6.540838	0.0239	
Residual	6.691981	13	0.514768			

R^2^ = 0.85, adjusted R^2^ = 0.77, predicted R^2^ = 0.16, C.V. % = 26.30, Adeq precision = 14.15.

According to the R^2^ statistic of 0.85, the model accounts for around 85.0% of the response’s variability. The model’s F-value of 11.77 implies that it is signed. There is only a 0.01% chance that an F-value this large could occur due to noise. A sufficient signal is indicated by the Adeq precision ratio of 14.152. As a result, this model is capable of navigating the design space.

**Figure 3:**
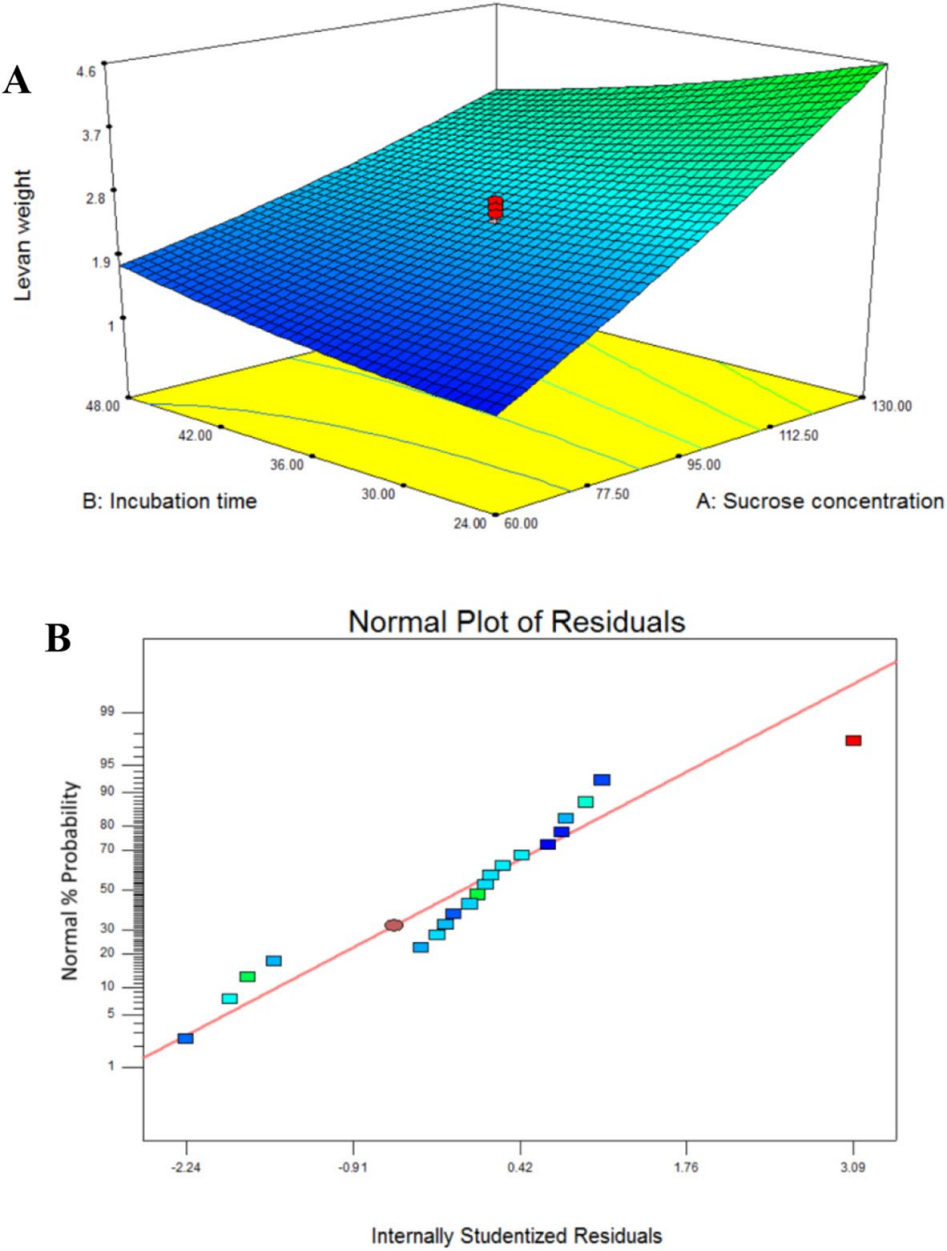
(**A**) (3D) response surface graph showing the significant interaction between levan and the two variables, sucrose concentration and incubation time while the other factor was held at zero level. (**B**) Normal plot of residuals emphasizing the model effectiveness where the points lie very close to the line.

The significant interaction between levan and the two variables, sucrose concentration, and incubation duration, is seen in Fig. 3A’s three-dimensional (3D) response surface graph, while the other factor was fixed at zero.

The final optimized medium constituents were g/L (130, sucrose; yeast, 1.0; K_2_HPO_4_, 0.25; MgSO_4_, 0.1) and gm/f (6.0, sugarcane bagasse; 1.0 banana peel). Incubation was 24 h, and the inoculation was 4 ml/flask of each (*Bacillus megaterium* OP315322) and (Rhizobium sp.OP363928). Sri Vinusha & Ganduri^50^ recorded sucrose and yeast extract concentrations (300 and 1.5 g/L respectively), and incubation time (132 h) as the factors that showed the maximum levan yield. On the other hand, Bouallegue et al.^9^ obtained the maximum yield at 162.5 g/L sucrose concentration and 72-h incubation.

It is important to note that banana peels are a waste product that is abundant in minerals and organic matter (lipids, fiber, carbohydrates, and protein)^50^_**.**_ The annual production of banana peels is around 36 million tons, and its existing endpoint is connected to negative environmental impact and financial losses^50^. The transformation of banana peel into a valued commodity would generate financial gain for the agriculture sector^51^.

#### Experimental validation of the model

The validity of the model was demonstrated by comparing the closely related statistically calculated yield for the overall design with the levan experimental yield. The normal plot of residuals also demonstrates the model’s effectiveness when the data points are close to a straight line (Fig. 3B). The optimization procedure has increased levan production. When compared to the non-optimized medium, the production on the optimized medium (81.50 g/L) nearly doubled. Using successive optimization designs, levan was generated by *B. subtilis* AF17 at a concentration of around 8 g/L by Bouallegue et al.^9^. However, Sri Vinusha and Ganduri^50^found that *Halomonas variabilis* MTCC ' maximal levan output was (63.31 g/L).

### Identification for the three levan types (G, K, M)

#### TLC and FTIR

**Figure 4:**
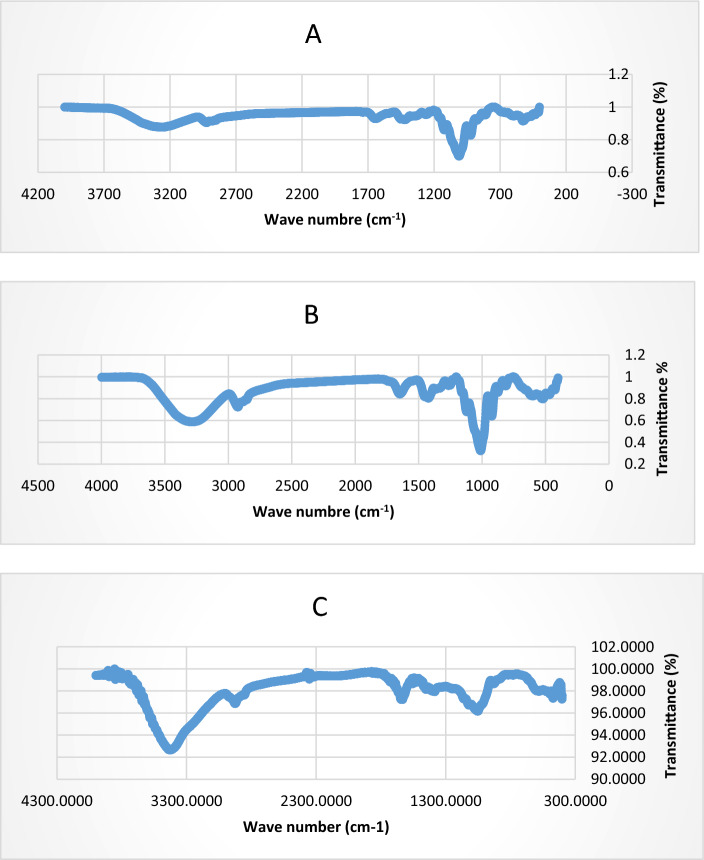
FT-IR transmission spectrum of the three levan types ((**A**), (**B**) and (**C**) represented G, K, and M respectively.

TLC results showed that fructose was the main backbone for the three levans (G, K, M). The resonant frequencies at which molecules can spin or vibrate are revealed by Fourier-transform infrared spectroscopy (FTIR), and these resonant frequencies are dictated by the nature of molecules with related vibronic coupling^52^. FTIR for the three levans types was used to determine the levan functional groups. G, K, and M showed broad stretching peaks at 3416.88.49, 3417.05, 3429.64 cm^−1^ respectively with variation degree. These peaks were pointed to the hydroxyl stretching vibration of the levan and it was noticed from Fig. 4A–C. that the spectrum of the coculture levan (M) was broader and stronger compared to the G and K. The broad absorption peak at 3390.40 cm^−1^indicated that the EPS sample contained massive hydroxyl groups (–OH) stretching vibration, which was a characteristic of polysaccharides^53^. The region among 900 cm^−1^ and 1132.17 cm^−1^ corresponded to fingerprints of polysaccharides^54^. G, K, and M recorded bands at 2920.96, 2979.55, and 2928.38 cm^–1^ respectively, which were attributed to C–H stretching vibration. The absorption band at 1648.18, 1642.23, and 1694.42 cm^−1^ for G, K and M represented the stretching vibration of the C=O carbonyl group. In addition, a weak symmetrical stretching represented the C–H bending was observed at 1422.93, 1420.71, and 1453.84 cm for G, K, M respectively. The C–H bending stretching vibration was attributed to an aliphatic CH2 group^50^.

At 1012.03, 1010.73, and 1051.28 cm the typical bending of C–OH was seen for G, K, and M respectively. This result ascertained the saccharide moieties existence^55^. The FTIR results concluded that there is a clear difference between the three levans in the hydroxyl region.

The coculture levan M showed a reduction in the hydroxyl group of phenolic which is the major compound in levan responsible for the bioactivity^56^. Also, the C=O group of flavonoid compounds of M appeared narrower in comparison to G and K. These groups play an essential role in the levan bioactivity. The stereochemistry of the hydroxyl groups in the pyranose ring can be significant for the production of molecules with endocyclic unsaturation^57^. Also, as seen in Fig. 4**. C** levan M appeared a great reduction in the C–OH region in comparison to the G and K.

#### ^13^C NMR

**Figure 5:**
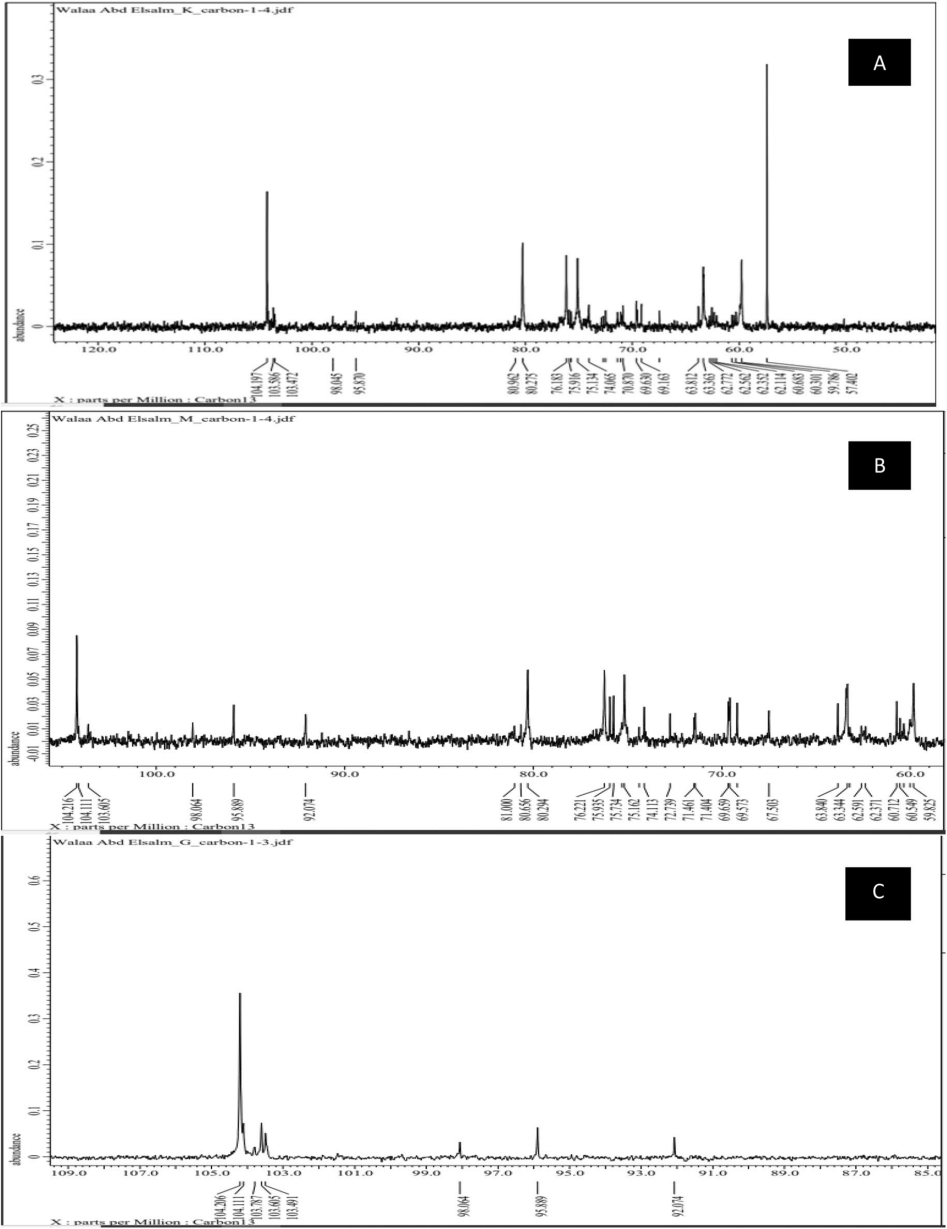
^13^C NMR of the three levans types ((**A**), (**B**) and (**C**) represented G, K, and M respectively).

^13^C NMR Spectrum was used to determine the number of distinct C atoms in a moleculeand analyze the configuration of the glycosidic bond in EPS. In ourstudy, ^13^C NMR of G, K, and M samples showed distinct characteristic signals of levan at δ104.206, 104.197 and 104.216 **(C-2)**, 80.99, 80.275, 80.294 **(C-5)**, 76.831, 76.183, 76.211, **(C-3)**, 75.925,75.935, 75.134 **(C-4)** respectively, 63.344,63.812, for G, K while M showed two characteristic δ at 63.840, 63.344 **(C-6)**, and 60.721, 59.786,60.721 **(C-1)** (Fig. 5A–C) The (β-2,6) linkage of the fructose moiety was confirmed by the downfield shift of C-2 and C-6.Also, Fig. 5A–C. revealed additional carbon signals at δ 95.889 and 92.047, in the case of G, K δ 95.87 and δ 95.889 for M pointed to free β-fructose, α and β free glucose. The signal at δ 98.064 in G, M, and 89.045 in K assumed glycoside linkage of glucose residue to the levan skeleton. In NMR investigations, signals in the region of δ 60 to 110 ppm were typical of polysaccharide spectra^58^. ^1^H NMR is extensively used for analyzing the configuration of the glycosidic bond in EPS.

#### ^13^C NMRused for analyzing the configuration of the glycosidic bond in EPS

##### ^1^H NMR

**Figure 6:**
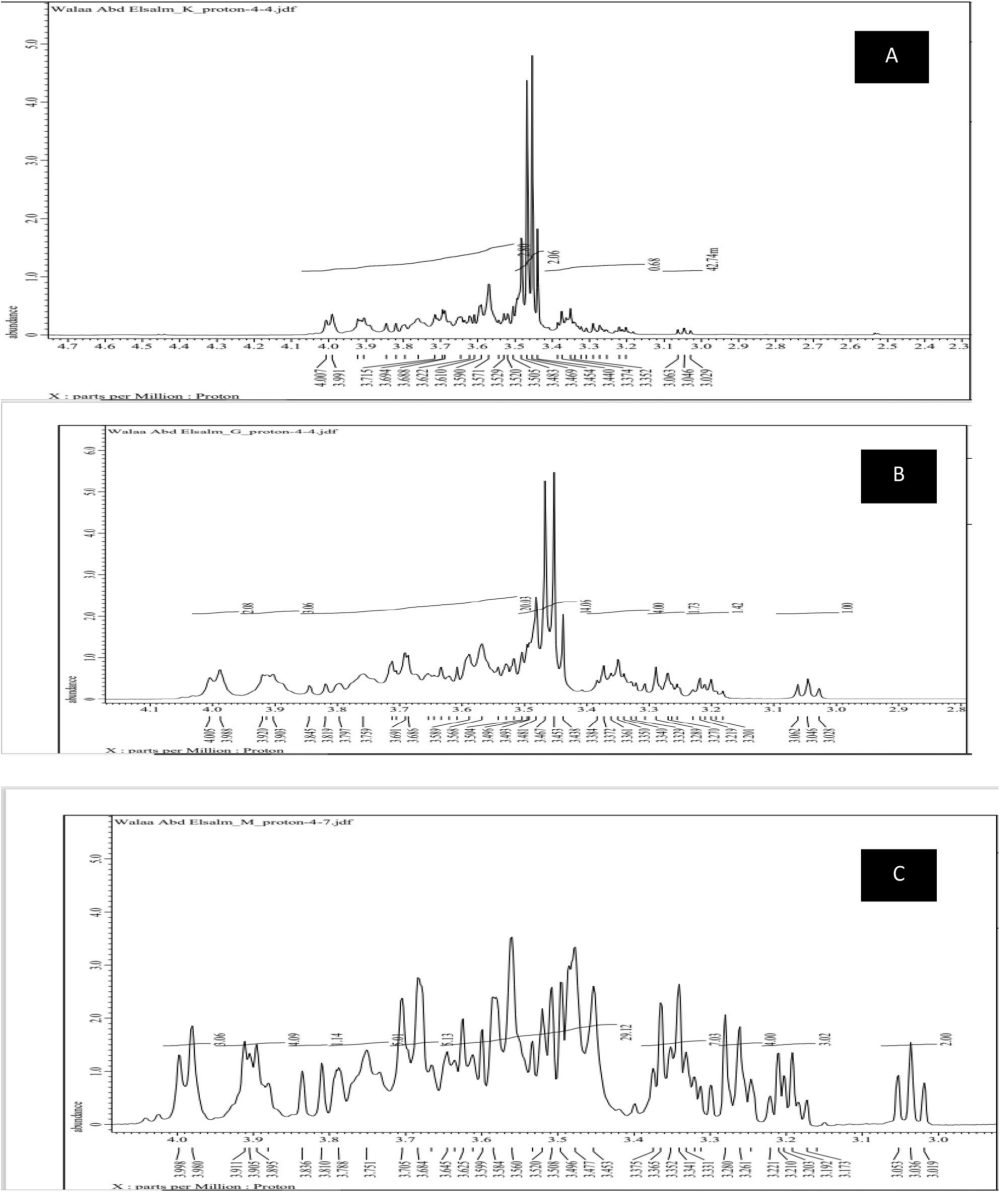
^1^H NMR spectra of the three levans types ((**A**), (**B**) and (**C**) represented G, K, and M respectively).

The three levan^1^H NMR spectra **(**Fig. 6A–C) displayed the levan distinct signals in the range of chemical shifts at 3.082 to 4.005 for G, 3.029 to 4.007 for K, and 3.019 to 3.998 for M. The cocultures levan (M) signals revealed a clear increase in signals in comparison to G, K. Wang et al.^58^ said that the signals at the 3.1–4.4 ppm region were back to C2–C6, but due to their significant overlapping it is hard to discern between them.

#### Molecular weight determination

The MW of the three levans G, K, and M were determined to be 1. 278 kDa, 9.088 kDa, and 11. 502 kDa, respectively. The results showed that the MW of M increased by about 9 and 1.16 compared to the MW of G and K, respectively. The increment of the MW of M could explain the difference in bioactivities between the three levans. Exopolysaccharides, particularly those that are homopolymers of EPS, have molecular weight-dependent activities^43^.

### Bioactivities studies in three levans types

In the following studies, the levan produced by (*Bacillus megaterium* OP315322, and *Rhizobium* sp. OP363928) and that produced by their coculture, M will undergo different characterization experiments.

#### Evaluation of prebiotic activity

The three kinds of levan (G, K, and M) showed remarkable prebiotic activity (Table 5). The levan type (M) recorded a superior promotion effect (63%) towards *L. plantarum* than the levan type (G, K) (46%). On the other hand, G and K levan did not affect *B. lichniformis* (88%), whereas M had a relatively comparable effect to the control. The superiority of M in comparison to G and K could be attributed to the strong and wide hydroxyl group of M. Cheng et al.^59^ reported a relative abundance of several beneficial bacteria, such as Eubacterium, and a significant increase in Anaerostipes, as well as a decrease in harmful bacteria like *Rumini Clostridium*, while no detectable change was recorded for Lactobacillus as a result of feed supplementation with levan produced by *Paenibacillus* sp. strain FP01.

**Table 5:** Prebiotic and antioxidant activities records of G, K, and M levan.

Levan type	Antioxidant activity	Prebiotic activity (%)	
		*Lactobacillus plantarum*	*Bifidobacterium lactis*
G	95.78	145.74	88.59
K	95.78	146.35	88.26
M	99.66	163.29	94.63

Generally, oligo-saccharides usually possess a great deal of prebiotic activity. Saleh et al.^60^ reported a variable promotion effect (18.75–25%) of xylooligosaccharides produced by *Aspergillus niger* MK981235 towards *L. plantarum*.

#### Antioxidant activity

The three types of levan (G, K, and M) scored a high level of scavenging activity (Table 5). Levan type M was superior to the other two types, it scored 99.66%. This result confirmed that the variation in the structure of the coculture M could play a role in the improvement of its activities, especially in the hydroxyl group region. Fructans may function as ROS detoxifying systems, offering an early line of protection against oxidative stress^61^. The highest DPPH scavenging activity of *Aspergillus niger* MK981235 fructooligosaccharides was recorded at 87.34%. Al-Qaysi et al.^62^reported that levan produced by *Pantoea agglomerans* scored 16.3% antioxidant activity at 200 μg/ml and reached the maximum, of 89.3% at 2 mg/ml.

The relative superiority of M in comparison to G and K could be attributed to its high MW. While the promising activity of G suggested backing to the broad hydroxyl and carboxyl groups. In the case of K the two factors proposed to affect the levan bioactivities)^6^. A wide stretching peak of O–H stretching for *Pantoea agglomerans* ZMR7 levan was observed at 3,417 cm^–1^. This type of levan showed different bioactivities, such as strong antioxidant, anticancer, and antiparasitic activities^62^.

#### Anti-migrating activity

**Figure 7:**
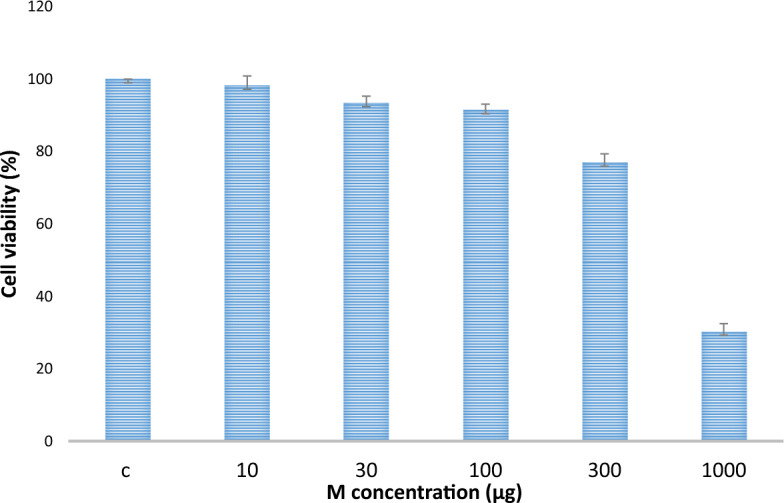
Anti-proliferative activity: the viability percentage of HepG2 treated with various concentrations of the coculture levan (M).

The result in Fig. 7 illustrated that the coculture levan (M) slowed epithelial wound healing compared to the untreated sample. It has long been understood that cancer and wound healing are related. It has been demonstrated that the processes that control wound healing encourage the proliferation and transformation of cancerous cells^63^.

#### Anticancer activity

**Figure 8:**
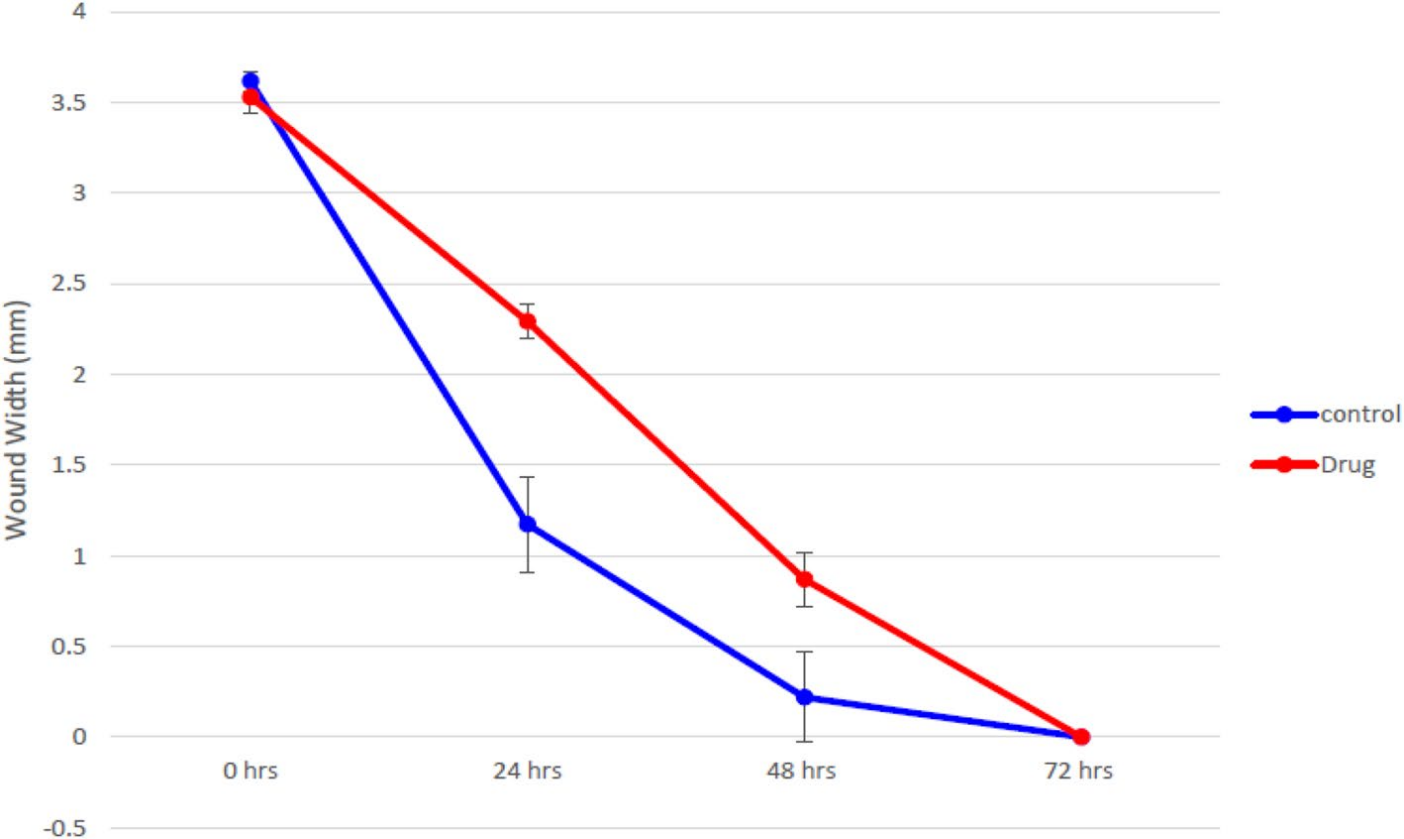
The effect of coulure levan (M) on the epithelial wound healing (B).

The results in Fig. 8 showed the cytotoxicity effect of levan different concentrations against HepG2. The results exhibited that the cell death increases with the increase of levan concentrations. The maximum cell viability inhibition (70%) was obtained at 1000µg. Abdel-Fattah, et al.^64^ reported that the *Bacillus subtilis* NRC1aza sulfated levan (SL) recorded high selective cytotoxicity against HepG2 cells.

### Gene expression in liver cancer cell lines (HePG2).

Gene expression analysis in liver cancer cell lines (HePG2) was performed using liver cancer-related genes such as CCL20, GRB2, and CCR6, as well as immune-related genes such as IL4R and IL10.

**Figure 9:**
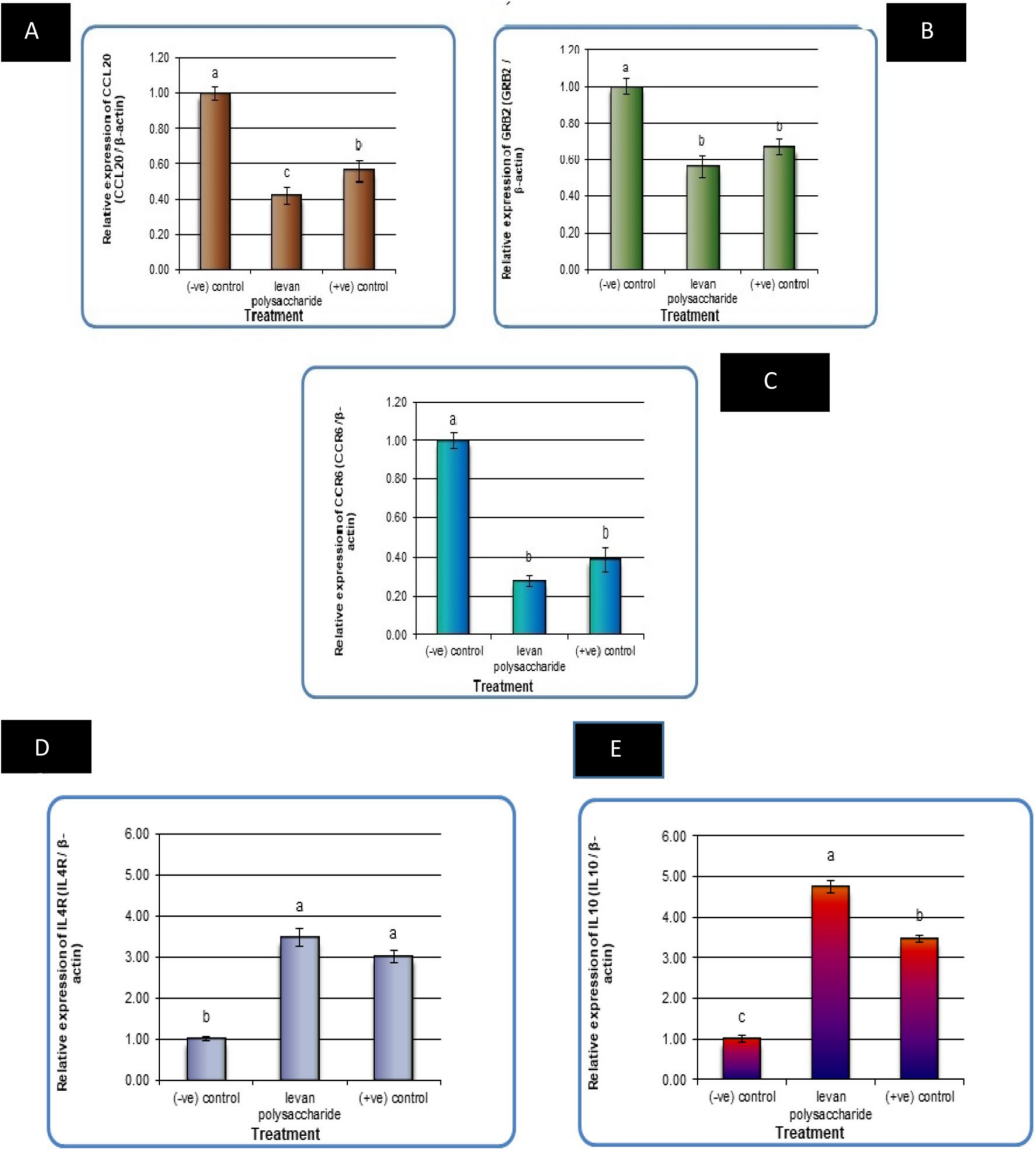
The alterations of CCL20 gene in liver cancer cell lines (HePG2) treated with 2% levan polysaccharide and Doxo (+ ve) control. Data are presented as mean ± SD. a,b,c: Mean values within tissue with unlike superscript letters were significantly different (P < 0.05) (**A**).The alterations of GRB2 gene in liver cancer cell lines (HePG2) treated with 2% levan polysaccharide and Doxo (+ ve) control. Data are presented as mean ± SD. a,b: Mean values within tissue with unlike superscript letters were significantly different (P < 0.05) (**B**). The alterations of *CCR6 gene* in liver cancer cell lines (**C**).The alterations of IL4R gene in liver cancer cell lines (HePG2) treated with 2% levan polysaccharide and Doxo (+ ve) control. Data are presented as mean ± SD. a,b: Mean values within tissue with unlike superscript letters were significantly different (P < 0.05). (**D**). The alterations of *IL4R gene* in liver cancer cell lines (HePG2) treated with 2% levan polysaccharide and Doxo (+ ve) control. Data are presented as mean ± SD. a,b,c: Mean values within tissue with unlike superscript letters were significantly different (*P* < 0.05) (**E**).

The results revealed that the expression levels of the CCL20, GRB2, and CCR6 genes were significantly increased (P 0.01) in negative samples of liver cancer cell lines compared with treated cell lines (Fig. 9A–C respectively). For the levan M group and (+ ve) control treated with Doxo, the expression levels of CCL20, GRB2, and CCR6 genes decreased significantly compared with the negative control. The expression levels of the CCL20, GRB2, and CCR6 genes were decreased in the levan polysaccharide group much more than those in the (+ ve) control group treated with Doxo without significant differences except for the CCL20 gene.

On the other hand, the expression levels of IL4R and IL-10 genes were significantly decreased significantly (P < 0.01) in negative samples of liver cancer cell lines compared with treated cell lines (Fig. 9D and E, respectively). For the Levan M group and (+ ve) control treated with Doxo, the expression levels of IL4R and IL-10 genes were significantly increased compared with the negative control. The IL4R expression levels and IL-10 genes were reduced in the levan polysaccharide group much more than those in the (+ ve) control treated with Doxo without significant differences except the IL-10 gene (Fig. 9D). It has been demonstrated that microorganism levan and plant 2,6-fructans can regulate the production of cytokines by immune cells in vitro^65^. The soil-bacterium *B. licheniformis* yielded a high-molecular-weight (Mw) levan EPS (2,000,000 Da) with β-2,1 branching. This bacterium causes the production of the pro-inflammatory cytokines IL-6 and TNF- by human whole blood cells^66^.

**Figure 10:**
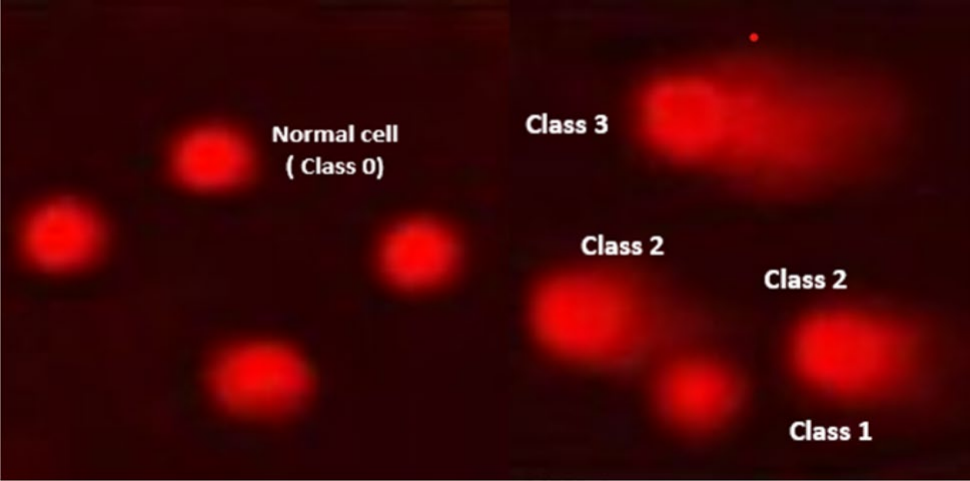
Visual score of normal DNA (class 0) and damaged DNA class 1, class 2 and class 3 using comet assay in liver cancer cell lines (HePG2).

The DNA damage in liver cancer cell lines (HePG2) was determined using a comet assay, as shown in Table 6 and Fig. 10. The results showed that negative samples of cancer cell lines exhibited a significant decline (P < 0.05) in DNA damage values compared with treated cell lines. However, the DNA damage values were significantly increased (P < 0.01) in treated liver cancer cell lines with levan polysaccharide and Doxo (+ ve control). The DNA damage values were significantly increased in the groups of levan polysaccharide and Doxo (+ ve control) in an ascending manner compared with the negative control. So, the DNA damage values were increased in the Doxo (+ ve control) group much more than those in the levan polysaccharide, but without significant differences. Abdel-Fattah, et al.^64^ reported that the *Bacillus subtilis* NRC1aza sulfated levan (SL) showed DNA damage and fragmentation that accompanied induced apoptosis via the mitochondrial pathway.

**Table 6:** Visual score of DNA damage in liver cancer cell lines (HePG2) treated with 2% Levan polysaccharide and Doxo (+ ve) control.

Treatment	No of samples	No. of cells		Class**				DNA damaged cells % (Mean ± SEM)
		Analyzed*	Comets	0	1	2	3	
(– ve) control	6	600	74	526	37	16	21	12.33 ± 0.88^b^
2% levan polysaccharide	6	600	145	455	48	41	56	24.17 ± 0.79^a^
(+ ve) control	6	600	161	439	52	37	72	26.83 ± 1.01^a^

*Number of cells examined per a group.

**Class 0 = no tail; 1 = tail length < diameter of nucleus; 2 = tail length between 1 and 2X the diameter of nucleus; and 3 = tail length > 2X the diameter of nucleus. Data are presented as mean ± SD.

^a,b^Mean values within tissue with unlike superscript letters were significantly different (*P* < 0.05).

## Conclusion

The study aims to achieve a basic point, which is to evaluate the coculture bacteria and their effect on the biological activities of levan through the use of a cheap medium for the scaling up of production that has economic returns. The results confirmed the superiority of bacterial cocultures in the productivity of levan, which is superior to single bacterial cultures, and also in the yield of M levan, which has better biological activity compared to the levan from single bacteria culture. The study introduced coculture techniques as a new way to get distinct types of levan.

## Data Availability

The datasets generated and/or analyzed during the current study are available in the GenBank repository, their accession numbers are (HepG2. BC020698.1, AF498925.1, AY242126.1, NG_012086.1 and HQ154074.1).
